# Short-read reading-frame predictors are not created equal: sequence error causes loss of signal

**DOI:** 10.1186/1471-2105-13-183

**Published:** 2012-07-28

**Authors:** William L Trimble, Kevin P Keegan, Mark D’Souza, Andreas Wilke, Jared Wilkening, Jack Gilbert, Folker Meyer

**Affiliations:** 1 Computation Institute, University of Chicago, Chicago, IL, 60637, USA; 2 Argonne National Laboratory, 9700 S. Cass Avenue, Argonne, IL, 60439, USA

**Keywords:** Gene prediction, Sequence errors, Short reads, Reading frames, Gene callers, Ab-initio gene prediction

## Abstract

**Background:**

Gene prediction algorithms (or gene callers) are an essential tool for analyzing shotgun nucleic acid sequence data. Gene prediction is a ubiquitous step in sequence analysis pipelines; it reduces the volume of data by identifying the most likely reading frame for a fragment, permitting the out-of-frame translations to be ignored. In this study we evaluate five widely used ab initio gene-calling algorithms—FragGeneScan, MetaGeneAnnotator, MetaGeneMark, Orphelia, and Prodigal—for accuracy on short (75–1000 bp) fragments containing sequence error from previously published artificial data and “real” metagenomic datasets.

**Results:**

While gene prediction tools have similar accuracies predicting genes on error-free fragments, in the presence of sequencing errors considerable differences between tools become evident. For error-containing short reads, FragGeneScan finds more prokaryotic coding regions than does MetaGeneAnnotator, MetaGeneMark, Orphelia, or Prodigal. This improved detection of genes in error-containing fragments, however, comes at the cost of much lower (50%) specificity and overprediction of genes in noncoding regions.

**Conclusions:**

Ab initio gene callers offer a significant reduction in the computational burden of annotating individual nucleic acid reads and are used in many metagenomic annotation systems. For predicting reading frames on raw reads, we find the hidden Markov model approach in FragGeneScan is more sensitive than other gene prediction tools, while Prodigal, MGA, and MGM are better suited for higher-quality sequences such as assembled contigs.

## Background

Next-generation sequencing technologies (reviewed in [1]) have dramatically reduced the per base cost of sequencing and, applied to metagenomics, have opened a new window into yet-uncultured organisms in the environment [2]. The ever-increasing rate of data generation, however, makes the processing and interpretation of large datasets increasingly expensive [3]. Running BLASTX against the approximately 4 billion amino acid NCBI nonredundant protein database on 1 Gbase of sequence requires approximately 10,000 [4,5] CPU-hours. Amazon on-demand extra-large EC2 instances at $0.68/hour put the computational cost of running BLASTX in the range of $7000 per Gbase. By analyzing sequenced DNA fragments and returning the coordinates and amino acid translations of ORFs that are likely coding regions, gene prediction can reduce the computational burden of protein similarity searches in metagenomic datasets by nearly a factor of 6.

Ab initio gene prediction tools (or gene callers) are currently used in popular metagenomic annotation pipelines. Methods for identifying genes in complete genomes (e.g., Glimmer [6] and GeneMark [7]) have been adapted for use on short fragments (such as MetaGeneMark (MGM) [8] and MetaGeneAnnotator (MGA) [9]). New algorithms have also been introduced, including Orphelia (OPH) [10], FragGeneScan (FGS) [11], and Prodigal (PRD) [12]. FGS is used in MG-RAST [13,14], FGS and MetaGene in CAMERA [15], MetaGeneAnnotator (MGA) in the annotation pipeline at the J. Craig Venter Institute (JCVI) [16], GeneMark and MGA in SmashCommunity [17], Orphelia in COMET [18], and a combination of tools including Prodigal, Metagene, MGM, and FGS in IMG/M [19-21]. The downstream processing of shotgun sequences in these pipelines uses various approaches to identify predicted protein fragments. MG-RAST uses BLAT against a protein database; CAMERA uses BLASTN against reference genomes; the JCVI pipeline uses a combination of BLASTP and protein hidden Markov models; COMET uses a machine-learning-based classifier UFO; and IMG/M and Smashcommunity use BLASTP. These all (except CAMERA) take advantage of the fact that ab initio gene calling is computationally inexpensive compared with the protein annotation step.

Table 1 lists the running times of the ab initio gene callers for 1 Gbase of sequence data on an Intel Xeon 2 GHz Linux server. Performing nucleotide-to-protein similarity searches against the 4 billion amino acid NR database requires 10,000 hours for BLASTX [4] or approximately 600 hours for RAPsearch [4]. A protein-only search would require an estimated 50 hours for BLAT [22]. Even the slowest ab initio gene callers take no more than 6 (FGS) and 13 (Orphelia) hours to process 1 Gbase. Since the time to apply even the slowest gene callers is still much smaller than the time required for downstream annotation by database searching, gene prediction is unlikely to be the limiting step in analysis of protein sequences from shotgun sequencing.

**Table 1:** Running times per gigabase of sequence data on a single 2 GHz processor

**Tool**	**Method**	**Symbol**	**Ref.**	**Time/Gbase**
FragGeneScan	Hidden Markov Model	FGS3,FGS5	[11]	6 hours
MetaGeneAnnotator	Codon usage + start site heuristics	MGA	[9]	15 min
MetaGeneMark	Codon usage + gc-content heuristics	MGM	[8]	20 min
Orphelia	Neural network	OPH	[10]	13 hours
Prodigal	Codon usage + dynamic programming	PRD	[12]	30 min

Compared with downstream analyses, ab initio gene calling is computationally inexpensive.

To be useful for large-scale sequence processing, gene callers must not utilize prior knowledge of the sequence environment—they must be one-size-fits all—and they must not require expensive computation such as a similarity search step. These requirements make self-training [23] and homology-based [20] gene callers suited for smaller-volume, higher-value annotation applications but not for raw reads.

The accuracy of gene prediction tools for short reads is limited by several factors, notably read lengths and sequencing errors. Read lengths (currently 100–250 bp for Illumina, 300 bp for IonTorrent, 500 bp for 454-pyrosequencer) are shorter than typical gene sizes (average 941 bp for the genomes used here) and may contain partial genes, gene boundaries, and sequencing errors. Error can vary widely as a result of sample quality and composition, sequencing preparation methods, vendor technology, and sequencing hardware maintenance. Vendor estimates of sequencing error range from 0.1 to 4% [24-28]. Further investigation suggests that the error range may be much higher [29]. While some fragments can capture entire short genes, in the regime where fragment lengths are shorter than typical gene sizes, most fragments will contain a single partial gene or fragments of two adjacent genes. Achieving good prediction performance on the largest likely gene fragment is the desired behavior for a read annotation system, since the largest fragment is both the most valuable and the strongest evidenced.

Previous studies have examined the accuracy of gene-calling algorithms to substitution errors typical for Sanger sequencing in 700 bp fragments [30] and the effect of insertion/deletion errors typical of Roche 454-pyrosequencing (~300 bp) [31], and have compared subsets of the current gene-calling programs [11]. Here we compare the performance of five gene-calling algorithms—FGS, MGA, MGM, OPH, and PRD—in the presence of varying rates of simulated sequencing error (where gold-standard annotations are available) as well as their performance on “real” metagenomic datasets.

## Results

### Accuracy in simulated data across varying error and fragment length

Detailed evaluation procedures are included in the Methods and Additional file 1 sections. In short, a single reading frame is identified at the center of each fragment as the “correct” reading frame if it is coding and the gene prediction tools are judged against this single correct answer for each read. The reading frame at the center of each fragment was calculated using the genome coordinates from which the fragment was taken and the genome coordinates of the first annotated gene overlapping the center of the fragment. Fragments whose center was not included within a gene were labeled explicitly as "noncoding". Thus each fragment was labeled with one of seven labels representing the "annotated reading frame". Sensitivity is the ratio of correctly predicted coding fragments to fragments annotated as coding; specificity is the ratio of correctly annotated noncoding fragments to fragments annotated as noncoding; and overall accuracy is an incidence-weighted combination of the two, explained more fully in the Methods section. Unlike some prior evaluations, this work explicitly counts gene predictions in the wrong reading frame as errors and explicitly defines and counts true negative (correctly predicted noncoding regions) predictions.

**Figure 1:**
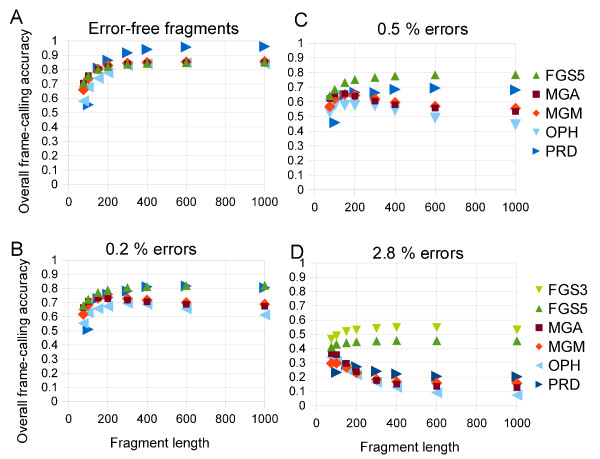
**Reading frame accuracy as function of fragment length for fragments at varying insertion/deletion error rates.** (**A**) Error-free fragments. (**B**) Fragments with 0.2% insertion/deletion errors. (**C**) Fragments with 0.5% insertion/deletion errors. (**D**) Fragments with 2.8% insertion/deletion errors. For error-free fragments, longer fragments result in more accurate predictions.

The overall accuracy of the five gene callers was determined on simulated shotgun sequences from fourteen prokaryotes as a function of fragment lengths between 75 and 1000 bp at four rates of artificially introduced insertion/deletion error (0%, 0.2%, 0.5%, and 2.8%). These error rates were selected for comparison with previous studies [19]. The overall accuracy is plotted against fragment length in Figure 1.

Applied to error-free fragments, all the tools have similar accuracy, and all the gene callers become more accurate with longer fragments, increasing from 60–77% for 75 bp fragments to 93–96% for 1000 bp fragments. Starting at insertion rates of 0.5%, where most reads have at least one error, the gene callers other than FGS demonstrate decreasing accuracy with increasing length. For long (400–1000 bp) fragments containing errors, gene callers make predominantly false-negative mistakes, failing to predict genes where annotated genes are present. Thus, current methods classify long, error-containing fragments as noncoding, most likely because of errors that induce frame shifts or generate spurious stop codons. This situation is problematic for metagenomic analysis because fragments that are incorrectly identified as noncoding are lost for further analysis.

For short fragments, the most common error is to call the gene on the reverse strand. Chargaff’s rule for oligomers [32,33], the observed similarity between the abundances of short nucleotide sequences and their reverse complements, may partly explain this type of error: this property makes the reverse complement of the correct frame have codon frequencies more similar to coding frames than other incorrect frames.

FragGeneScan is more accurate than the other four methods at predicting the correct reading frame in fragments with error rates above 0.5%. The differences in accuracy in the presence of errors are small for short (<150 bp) sequences but become as great as 25% for long (>400 bp) fragments with errors. Orphelia, by contrast, showed lower overall accuracies than did the other four methods, particularly in the presence of substitution errors. Prodigal showed poor performance for fragments shorter than 200 bp.

The overall accuracy, sensitivity, specificity, and positive predictive value figures for four previously published benchmark datasets with varying insertion/deletion rates are reported in Table 2, and for five datasets with varying substitution rates in Additional file 2: Table S1. Using the 0.2% dataset as an example, FGS correctly labels only 59.7% of noncoding sequences, whereas MGA, MGM, and Prodigal have specificities of 85.2%, 84.1%, and 69.4%, respectively. The overall accuracy as a function of error rate for the benchmark datasets is plotted in Additional file 3: Figure S1, showing that insertion and deletion errors cause loss of accuracy faster than do substitution errors, which do not alter the reading frame.

**Table 2:** Accuracy, sensitivity, specificity, and PPV for benchmark datasets with simulated 454-style errors

**Overall reading-frame accuracy**					
**Dataset**	**FGS**	**MGA**	**MGM**	**OPH**	**PRODG**
0.00%	91.0%	93.6%	94.5%	90.5%	91.7%
0.20%	87.8%	81.3%	82.3%	76.0%	78.9%
0.50%	83.8%	69.7%	70.5%	62.9%	66.1%
2.80%	58.4%	25.9%	26.0%	22.1%	23.2%
Sensitivity					
0.00%	95.2%	94.7%	95.9%	92.8%	95.0%
0.20%	91.5%	80.8%	82.1%	76.3%	80.7%
0.50%	87.1%	67.7%	68.6%	61.3%	66.5%
2.80%	59.7%	18.3%	18.2%	15.0%	19.4%
Specificity					
0.00%	59.0%	84.2%	82.9%	71.8%	68.5%
0.20%	59.7%	85.1%	84.0%	73.3%	66.7%
0.50%	58.8%	85.9%	85.6%	75.5%	66.4%
2.80%	49.0%	89.2%	89.8%	81.5%	65.8%
Positive Predictive Value					
0.00%	91.6%	96.1%	96.4%	93.2%	94.0%
0.20%	88.9%	90.9%	91.2%	86.5%	86.6%
0.50%	85.5%	86.0%	85.9%	80.2%	78.1%
2.80%	62.1%	58.0%	56.5%	44.0%	35.4%

**Figure 2:**
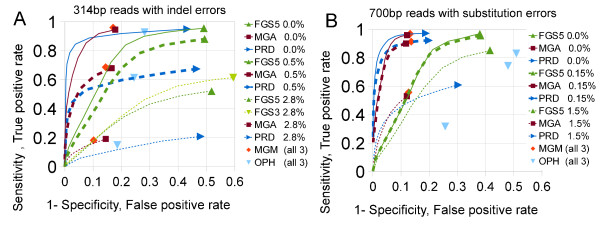
**Receiver operating characteristics for three gene callers at varying rates of error.** (**A**) Three rates of insertion/deletion error in 317 bp fragments. (**B**) Three rates of substitution-error in 700 bp fragments. Colors and symbols indicate gene callers; line style (solid, dashed, dotted) indicates dataset simulated error rate. The default operating point is the rightmost point on each graph. Metagenemark and Orphelia do not output confidence scores; consequently their performance is indicated by only one point per error rate dataset.

The relationship between sensitivity and the specificity at different thresholds for gene identification can be calculated for three of the gene callers (FGS, MGA, and Prodigal) that give scores to each predicted gene, where lower false-positive rates can be explored by applying more stringent thresholds than default to the gene predictions. Graphs of sensitivity vs. specificity (receiver operating characteristic curves) [34] are given in Figure 2 for varying rates of introduced error, both insertion/deletion and substitution, These show a clear tradeoff in the choice of tools; for error-free data, Prodigal and MGA significantly outperform FGS, offering comparable sensitivity at lower false-positive rates. But at rates of 0.5% insertion/deletion errors and 1.5% substitution errors, Prodigal and MGA miss 40% of the genes that are present.

The better sensitivity of FGS in error-containing, short-read data comes at the cost of lower specificity—FGS is less able to recognize non-coding regions—and predictions of genes that are longer than the Refseq annotations. FGS translates noncoding regions as coding and tries to correct pseudogenes by inferring frameshifts as erroneous insertions and deletions.

Unlike the other four tools, FGS explicitly predicts probable insertion and deletions in individual fragments; these result in longer (though not always accurate) predicted coding regions. The other methods report multiple predicted gene fragments when they find conflicting reading frame evidence. FGS, because of its algorithm, is forced to choose between overlapping potential genes and cannot issue overlapping predictions.

**Figure 3:**
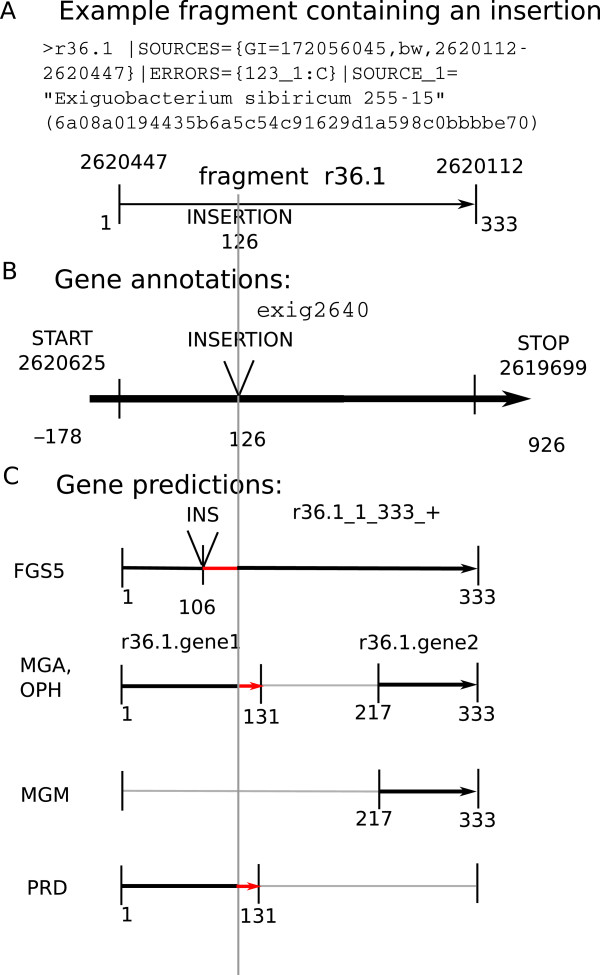
**Example fragment containing an insertion.** (**A**) The fasta header for an error-free artificial fragment from E. sibricum that contained a single, artificial insertion near the center of the fragment. This insertion disrupted gene prediction in all five gene callers. (**B**) Refseq annotations show this fragment is entirely contained within one annotated gene, though it is artificially split into two reading frames. (**C**) Fraggenescan predicts an insertion in the wrong place, leading to seven nonsense amino acids adjacent to the insertion. The other gene prediction tools predict one or two shorter fragments, one of which has nonsense residues at the end of the prediction. The alignment-based evaluation technique would count all five as true positives because of the length of the correctly translated regions; the reading-frame technique would count all but FGS as false negatives because of their failure to correctly translate the middle of the fragment.

The contamination of predicted genes with nonsense protein sequence is an inevitable consequence of uncorrected sequencing errors, and the gene callers treat this issue in different ways. Figure 3 shows an artificial fragment containing an insertion that disrupts gene calling and causes all of the gene callers to miss at least some of the correct amino acids. Fraggenescan predicts the insertion in the wrong place, leading to seven out-of-frame peptides adjacent to the insertion. The other gene prediction tools do not attempt to predict insertions, and all either predict nonsense amino acids at the end of predicted gene1 (MGA, OPH, PRD) or miss most of the coding sequence (MGM). This illustrates the main consequence of the difference between FGS and the other tools—FGS’s predictions are longer, more sensitive, and contain more nonsense than those of the other tools.

It is in principle possible to infer the presence of frame shifts in the output of MGA, MGM, and Prodigal by recognizing adjacent or overlapping reading frames and guessing that a frameshift has occurred [35]. With additional evidence from an alignment to a reference sequence or model, the location of frame shifts can be positioned more precisely. This technique has been to correct frameshift errors in reads with alignments [36].

### Coding fraction profiles on real metagenomic data

To investigate the behavior of the current generation of ab initio gene callers on real metagenomic data, we applied all five gene callers to three shotgun sequencing datasets that span three next-generation sequencing technologies in two medium-complexity metagenomic environments: one from cow rumen [37] and two from distal human gut [38].

**Figure 4:**
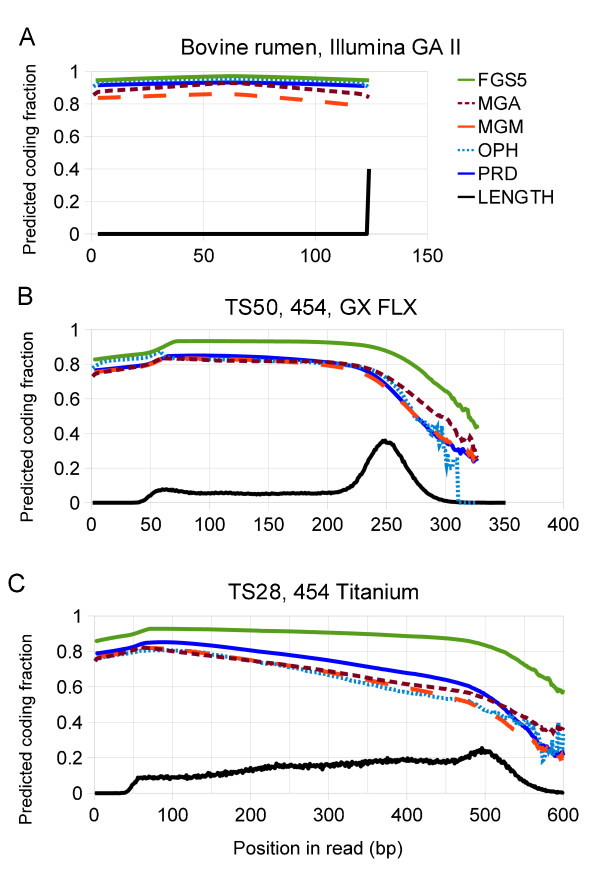
**Predicted coding fractions as a function of position in read for three metagenomic datasets.** The black lines are proportional to the read-length histograms. All the gene predictors predict fewer genes at the end of fragments. Compare with Additional file 3: Figure S2.

The predicted coding fraction, defined here as the fraction of the reads at least n bases long that have the *n*th base contained within a predicted gene [31], is plotted in Figure 4 as a function of position in the read for these three datasets for each of the five gene callers. MGA, MGM, Orphelia, and Prodigal all predict similar coding fractions for all the datasets, while FGS predicts higher coding fractions. All the programs predict high coding fractions (85–95%) for the shortest (Illumina) reads at 125 bp and smaller coding fractions for the longer (250 and 500 bp) 454 reads. A surprising result is that for the dataset generated by using the Roche-454 pyrosequencing Titanium platform, all the gene callers (except FGS) predict a coding fraction that decreases from 80% at the beginning of the fragment to 50% at the modal sequence length of 500 bp. This result is consistent with the expectation that error rates increase with position in pyrosequencing reads [28]. This decreasing coding fraction suggests that sequencing errors may be disrupting the identification of genes near the end of the fragments. It is reasonable to expect that quality filtering [25] and quality-aware read trimming [29] before gene calling will improve accuracy of the predicted translations.

Artificial datasets containing insertions and deletions did not have a pronounced change in the predicted coding fraction between the beginning and end of the reads except at the highest substitution error rate tested (1.5%). Additional file 3: Figure S2 shows coding fraction profiles for benchmark datasets with known errors for comparison with the metagenomic datasets. Since the datasets simulate shotgun data, the distribution of “real” annotated genes in the fragments is uniform over the fragment length. For longer (>400 bp) fragments with errors, MGA and MGM show lower densities of predicted genes in the center of the fragment than at the ends, suggesting that the enumeration of open reading frames (which assumes that the stop codons are real) results in biases in gene calling that depend on position within the read.

## Discussion

The exclusive use of sensitivity and positive predictive value (PPV) [11] in training and describing the accuracy of gene-calling tools has had unintended effects on their development [39]. Current tools accurately identify noncoding regions but are poorly equipped to handle data containing sequencing errors, even at the relatively modest levels reported by technology vendors.

To quantify gene prediction accuracy, the gene detection literature has used sensitivity and specificity for whole genes [6,7,30], reading-frame-aware sensitivity and specificity [8], alignment-based sensitivity and PPV [11] and amino acid sensitivity [31]. Some of these metrics penalize false-positive and false-negative predictions essentially equally. We find that reading-frame-aware, prospective sensitivity agrees with amino acid sensitivity better than with per fragment alignment-based sensitivities on the same datasets.

The frequency and impact of inaccurate gene calls are relatively low in noncoding DNA. Combinations of sensitivity and specificity that weigh the errors according to their expected number are an effective way to gauge prediction accuracy while utilizing the assumption that most (85–90%) [40] of the sequence in prokaryotic genomes is annotated as coding. Such an expected-incidence combination was introduced as “prediction accuracy” [39], but the testing dataset used was engineered to have specific gene boundaries and had only a 50% coding fraction as a result. When overall accuracy is used on datasets engineered to mimic shotgun data [31,41], the results are close reflections of sensitivity.

The observation that at high error rates increasing fragment length does not improve gene prediction accuracy is instructive. Sequencing errors, particularly frameshift errors, dilute the evidence for coding regions by spreading the signal among competing adjacent reading frames. Since only bases without an interrupting error can contribute in the correct frame, increasing length will improve accuracy only until the length well exceeds the mean distance between errors, twice the reciprocal error rate. For fragment lengths below 100 bp and error rates above 2%, reading-frame prediction accuracy is poor. This argues against applying ab initio gene callers unless read error rates can be pushed below 2%. For the PacBio systems [42] platform, which offers raw reads >3 kb at error rates of 15% and circular template corrected reads at 400 bp with error rates <1%, ab initio gene prediction can be expected to work on the corrected reads but fail on the uncorrected reads despite their length.

## Conclusions

When annotating individual reads, sequencing errors cause a loss of predicted coding regions, leading to loss of signal. FragGeneScan exhibits superior sensitivity in error-containing reads with respect to reading frame prediction, and tends to over-predict nonconcoding regions as nonsense. MGM, MGA, and Prodigal offer accurate predictions as long as reads are error-free. Prodigal performs somewhat better than MGM and MGA on error-free data.

The evaluation procedure for the algorithms to predict genes inevitably guides the future progress of gene prediction tools. Treating reading-frame prediction as a binary classification problem leads to overestimation of the accuracy of the programs and tuning of these programs to accurately identify noncoding regions. For this reason, evaluation schemes that explicitly test reading frame and those that count aligned amino acids are preferred over methods that count only the number of alignments found.

Sequencing technologies at present can produce multiple billions of reads as long as 250 bp [43,44], a length regime where prokaryotic gene prediction accuracy is around 90%. Protein annotation steps remain computationally expensive and stand to become even more so as the size of reference databases grow. Ab initio gene calling trades accuracy for speed. The annotation pipelines for raw reads have taken this tradeoff—a factor-of-6 cheaper annotation in exchange for missing perhaps 10% of the genes that are there. It is likely that the experimenter’s choice of sequencing methods will be dominated by length limitations in other stages in the processing of metagenomic data, such as similarity searching, where fragments shorter than 400 bp show diminished sensitivity [45].

## Methods

### Datasets

The three metagenomic datasets were downloaded from the NCBI sequence read archive http://www.ncbi.nlm.nih.gov/sra as accession numbers SRR029690 (MGR:4440613.3) for TS28, SRR029697 (MGR:4440615.3) for TS50, and SRR094166 (MGR:4465811.3) for rumen. For the rumen dataset only the first 500,000 reads were used.

Genome sequences and RefSEQ annotations for the sequenced organisms were downloaded from NCBI http://www.ncbi.nlm.nih.gov/genbank[46].

For evaluating gene-calling accuracy as a function of fragment length, datasets were created with specified fragment lengths using Metasim [47] using the set of fifteen prokaryotic chromosomes listed in [30], also listed in Additional file 2: Table S2. From each chromosome 5,000 fragments were generated at each of the lengths 75, 100, 150, 200, 300, 400, 600, and 1000 base pairs using the model parameters described in [31] to generate similar error rates.

For comparison with other evaluations, the nine benchmark sets described in [31] were downloaded from http://metagenomic-benchmark.gobics.de. The datasets, their sizes, and their exactly measured error rates are listed in Additional file 2: Table S3. Four datasets have insertion and deletion error rates of 0, 0.2%, 0.5%, and 2.8% on fragments of length centered at 315 bp; and five datasets have mostly substitution errors at rates of 0, 1.5 × 10^−5^, 1.5 × 10^−4^, 1.5 × 10^−3^, and 1.5%, with fragments of lengths centered at 700 bp.

### Evaluation

Since the sequence fragments used for evaluation here are artificial, the genome and genome coordinates from which each read is derived are exactly known. Each fragment was labeled as noncoding or with the reading frame corresponding to the center of the fragment using the genome coordinates from which the fragment was taken and the genome coordinates of the first annotated gene overlapping the center of the fragment. This reflects two decisions to define a single correct answer for each fragment—the choice of the center of the fragment, which emphasizes accuracy at the place in the read where the evidence is strongest, and an arbitrary choice to resolve annotations that are ambiguous as to the correct reading frame. For reads containing simulated insertions or deletions, the cumulative number of simulated insertions and deletions between the beginning of the fragment and the center of the fragment must be used to adjust the predicted reading frame (referenced to the beginning of the read) to the real reading frame (after the introduction of artificial insertions and deletions). Failing to apply this correction leads to the appearance of decaying reading frame accuracy with increasing fragment length for reads containing indels, since the naively calculated reading frame is only correct between the beginning of the read and the first (artificially-introduced) indel.

Following the procedure of [31], the overall results presented for all the artificial datasets are averages of the performance metrics over the fourteen prokaryotic species used, where species receive equal weight.

The outputs of the gene callers were filtered for gene predictions that included the coordinate of the center of each fragment. The reading frames were normalized to a common format; and the numbers of fragments with each annotation and each prediction were sorted into 49 categories, indexed by true and predicted reading frame. Sums of elements in this matrix (described in Additional file 1) give the categories of correctly labeled coding regions (TP), correctly labeled noncoding regions (TN), incorrectly labeled coding regions (FP), and false-negative (FN) gene calls.

The performance statistics are defined conventionally [39], with the exception that genes that are predicted as genes but with the wrong reading frame form a nonoverlapping set of incorrect reading-frame assignments (WF) that enters in the denominators: Sn = TP/P = TP/(TP + FN + WF), Sp = TN/F = TN/(TN + FP), PPV = TP/(TP + FP + WF), where N_tot_ = TP + TN + FP + FN + WF.

## Abbreviations

FGS: FragGeneScan; FN: False negative; FP: False positive; JCVI: J. Craig Venter Institute; MGA: MetaGeneAnnotator; MGM: MetaGeneMark; NC: Non-coding; OPH: Orphelia; PRD: Prodigal; TN: True negative; TP: True positive; WF: Wrong-frame assignment.

## Competing interests

The authors declare that they have no competing interests.

## Authors’ contributions

WT, KK, and FM conceived and designed the study; MD AW and JW guided the technical implementation and evaluation. WT wrote and JG helped edit the manuscript. All authors have read and approve of the manuscript.

## Supplementary Material

Additional file 1 Supplemental Methods. Click here for file

Additional file 2 Supplemental Tables. Click here for file

Additional file 3 Supplemental Figures. Click here for file
