# Supplementary material for: Short-read reading-frame predictors are not created equal: sequence error causes loss of signal
Source: BMC Bioinformatics. 2012 Jul 28;13:183. doi: 10.1186/1471-2105-13-183 (PMC3526449; doi:10.1186/1471-2105-13-183)
Supplement: Additional file 1 — Supplemental Methods. [file 1471-2105-13-183-S1.doc]

**Supplemental Methods:**

***Reading-frame-aware accuracy metrics***

Gene calling accuracy has traditionally been reported by using ratios of the numbers objects in sets classified as correct or incorrect, treating gene calling as a binary classification problem . Initially adapted from work annotating complete genomes , these were at first ratios of the numbers of genes that were predicted to the number of genes that were annotated. Some authors defined a true positive as a gene present “anywhere in the read where a gene is ex­pected” , judging entire fragments based on gene or nongene annotation status. Other authors have imposed more stringent standards that require at least part of the prediction to be in the correct read­ing frame, either by calculation of the reading frame (“match between predicted and annotated reading frames” ) or by the existence of at least one alignment of sufficient length, typically 20 amino acids, to the annotated protein sequence . We note that reading-frame-aware evaluations are not strictly comparable to (older) evaluations using only gene/nongene status.

The alignment-based evaluation procedures count the number of annotated gene fragments, the num­ber of predicted gene fragments, and the number of subsets of each of these two that are “correct” as revealed by protein alignments between the predictions and the annotations. True positives (TP) are pre­dictions that contain a (correct reading frame) alignment, false positives (FP) are predictions that lack an alignment, and false negatives (FN) are annotations for which there is no corresponding prediction. True negatives (TN), nongenes that are correctly identified as such, are not defined by using the alignment-based method because fragments with no annotation are not considered.

Yok and Rosen have pointed out that the ab initio gene-calling literature has used a definition of “specificity” that corresponds to positive predictive value (the ratio of true positives to annotated positives, PPV =(TP )/(FP + TP )) in place of the traditional specificity Sp = T N/(TN + FP) . This is partly a consequence of the absence of the true negative category in alignment-based evaluation.

***Character of prediction data***

The number of predicted gene fragments is in general not equal to the number of reads or the number of annotated fragments; this situation complicates the construction of the sets of TP and FP. One read can overlap multiple adjacent genes, and gene-calling algorithms can predict multiple gene fragments in a single read. Additionally, more than one prediction can correspond to different parts of the same annotation (in the case of a frame shift), and one predicted gene can span multiple annotated ones (if a stop codon was mistaken for a frame shift). Consequently, researchers using alignment-based evaluations have needed to define two types of true positive—true positives corresponding to unique annotations (for calculating sensitivity) and true positives corresponding to unique predictions (for calculating PPV).

Figure S3 shows a fragment that has two annotations and two gene predictions from all the tools. Both of the annotated genes that overlap this fragment have protein similarities to the predicted genes, so the alignment-based technique counts this read as two true positives. By contrast, the reading-frame technique introduced here counts the read as a single true negative because there are no annotated genes in the center coordinate of the fragment and there are no predictions in the center of the fragment. For this fragment, which is error-free, all five algorithms gave identical, correct predictions.

***Confusion matrix***

The ambiguities as to the number of objects being evaluated can be resolved by assigning a single label with the annotated reading frame to each fragment, creating one correct answer per fragment. Comparing the label(s) assigned by the gene predictors with the annotated label is then straightforward. Unlike the alignment-based approach, this procedure defines true negatives, the four sets do not overlap, and the sum of the categories is the number of fragments that are being tested.

The predictions for annotated fragments were counted and arranged in a 7 × 7 matrix. We follow the convention that reading frames 1–3 are those on the forward strand, 4–6 are on the reverse strand, and the index 7 represents noncoding.

Sums of the elements in this confusion matrix give the classical categories.


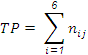


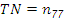


Here *n_ij_* represents the number of fragments that were annotated with reading frame j that were labeled with reading frame *i*. False negatives are the sum over six matrix elements of misidentified noncoding regions.


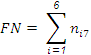


The false-positive category covers the six elements that are incorrectly labeled as coding.


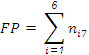


A catch-all category, not present in the binary classification system, exists for genes that are predicted as genes but in the wrong frame. These are counted as errors.


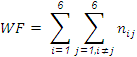


The confusion matrix is drawn in Figure S4 to highlight these categories; representative results comparing FGS to MGA are in Figure S5.

To characterize the performance of gene callers for sequence fragments, we initially examined basic properties of the predictions for nine previously published “benchmark” datasets . Four of the datasets have lengths of 315 bp and have different rates of insertion/deletion errors. Five of the datasets have 700 bp average lengths and have varying rates of error that is mostly substitution. For these nine benchmark datasets, the numbers of predicted genes, the predicted coding fractions, and the average predicted gene lengths are presented in Tables S4 and S5.

***Comparisons***

Our accuracy results are in essential agreement (always within 4%) with prior accuracy investigations on error-free fragments and error-containing fragments where performance numbers are comparable. Prior assessments have tended to use overlapping but nonidentical sets of genomes for testing; these are listed in Table S2.

The reading frame sensitivity and positive predictive values in this evaluation are in general agreement (the differences are less than 5%) with the amino acid sensitivity and amino acid PPV previously evaluated for these datasets . The reading frame metrics do not agree as well, however, with the alignment-based sensitivity and PPV values. At high error rates, the overall reading frame accuracy is much smaller than the alignment-based sensitivities and is in better agreement with the amino acid sensitivity.

We find positive predictive values somewhat higher (96% vs. 84% for MGA with error-free frag­ments) and specificities considerably higher (83% vs 42%) than those reported in . Since the difference between our results is large compared with typical (10–20%) interspecies differences in the gene-calling performance , we suspect that the large (50%) fraction of noncoding regions in the dataset of Yok and Rosen causes this disagreement.

***Software***

Orphelia was downloaded as a Linux executable from http://orphelia.gobics.de/download.jsp, MGA as a Linux executable from http://whale.bio.titech.ac.jp/metagene/download mga.html, and MetaGeneMark v1 as a Linux executable from http://exon.biology.gatech.edu/metagenome/Prediction in September 2010. FragGeneScan1.15 was downloaded as C source code from http://omics.informatics.indiana.edu/FragGeneScan. Prodigal 2.50 was downloaded from http://code.google.com/p/prodigal/.

The command-line parameters used for the gene predictors are listed in Table S6. FGS was tested with the 454 3% error model on the highest error (2.8%) insertion/deletion set, and with the Illumina ­0.5% error model otherwise. Orphelia was tested with its Net300 model for datasets with average lengths shorter than 300, and with Net700 otherwise. The running time comparisons for gene callers were done using TS28.

For the generation of testing data with defined lengths, MetaSim 0.9.1 was downloaded from

<http://www-ab.informatik.unituebingen.de/data/software/metasim/>download/welcome.html

using the error model parameters from . The clone length parameter was set to lengths of 75, 100, 150, 200, 300, 400, 600, and 1000. The width of the clone length distribution was set to zero. The datasets generated for this paper and the reading frame calls are included in MG-RAST project MGP1159, available at <http://metagenomics.anl.gov/linkin.cgi?project=1159>
