# Supplementary material for: Short-read reading-frame predictors are not created equal: sequence error causes loss of signal
Source: BMC Bioinformatics. 2012 Jul 28;13:183. doi: 10.1186/1471-2105-13-183 (PMC3526449; doi:10.1186/1471-2105-13-183)
Supplement: Additional file 2 — Supplemental Tables. [file 1471-2105-13-183-S2.doc]

Table S1. Accuracy, sensitivity, specificity, and PPV for benchmark datasets with simulated Sanger-style errors. The overall reading-frame accuracy drops for all the gene callers at high substitution error rates.

| Overall reading-frame accuracy | | | | | |
| --- | --- | --- | --- | --- | --- |
| Dataset | FGS | MGA | MGM | OPH | PRODG |
| 0.00% | 92.9% | 94.8% | 95.7% | 79.7% | 95.5% |
| 1.50E-05 | 93.0% | 94.7% | 95.6% | 79.9% | 95.2% |
| 1.50E-04 | 92.8% | 94.4% | 95.2% | 79.2% | 94.8% |
| 1.50E-03 | 91.8% | 89.7% | 90.8% | 72.2% | 90.8% |
| 1.50E-02 | 81.6% | 57.5% | 59.1% | 36.6% | 62.1% |
|  |  |  |  |  |  |
| Sensitivity | | | | | |
| 0.00% | 96.3% | 95.6% | 96.8% | 83.0% | 97.1% |
| 1.50E-05 | 96.4% | 95.6% | 96.8% | 83.1% | 97.1% |
| 1.50E-04 | 96.1% | 95.0% | 96.1% | 82.5% | 96.5% |
| 1.50E-03 | 95.2% | 89.9% | 91.2% | 74.3% | 92.1% |
| 1.50E-02 | 83.8% | 53.5% | 55.7% | 31.8% | 60.6% |
|  |  |  |  |  |  |
| Specificity | | | | | |
| 0.00% | 66.6% | 87.9% | 86.8% | 49.1% | 82.8% |
| 1.50E-05 | 66.4% | 87.3% | 86.2% | 49.3% | 81.8% |
| 1.50E-04 | 68.0% | 88.9% | 87.0% | 48.4% | 83.0% |
| 1.50E-03 | 66.0% | 88.0% | 86.4% | 52.0% | 81.6% |
| 1.50E-02 | 64.4% | 89.5% | 87.3% | 74.3% | 77.5% |
|  |  |  |  |  |  |
| Positive Predictive Value | | | | | |
| 0.00% | 93.6% | 97.4% | 97.4% | 87.0% | 97.1% |
| 1.50E-05 | 93.6% | 97.4% | 97.4% | 87.4% | 97.0% |
| 1.50E-04 | 93.6% | 97.3% | 97.2% | 86.8% | 96.9% |
| 1.50E-03 | 92.6% | 95.5% | 95.2% | 83.1% | 94.7% |
| 1.50E-02 | 84.7% | 83.8% | 81.6% | 63.5% | 76.8% |

Table S2. Genomes used for testing in previous benchmarking studies. Previous benchmarking studies have seldom used the same set of organisms. The studies of Zhu et al. , and Yok and Rosen , used sets of 50 and 96 organisms, respectively. This study uses datasets published by Hoﬀ in the benchmark datasets and the set of organisms used by Noguchi et al. for the study of length dependence.

| Name | Phylum | Accession | Bessemer, 1999 | Nogouchi, 2006 | Hoff, 2009 | Rho, 2010 | Hyatt, 2010 | Mb | Dom. |
| --- | --- | --- | --- | --- | --- | --- | --- | --- | --- |
| N. pharonis | Euryarchaeota | NC 007426 |  | * | * |  | * | 2.60 | A |
| A. fulgidus | Euryarchaeota | NC 000917 | * | * |  |  |  | 2.18 | A |
| M. jannaschii | Euryarchaeota | NC 000909 |  | * |  |  |  | 1.66 | A |
| M. thermautotrophicus | Euryarchaeota | NC 000916 | * |  |  |  |  | 1.75 | A |
| H. salinarum | Euryarchaeota | NC 010364 |  |  |  |  | * | 2.00 | A |
| Aeropyrum pernix | Crenarchaeota | NC 000854 |  |  |  |  | * | 2.69 | A |
| B. aphidicola | Proteobacteria (b) | NC 002528 |  | * | * | * |  | 0.64 | B |
| B. pseudomallei chr.1 | Proteobacteria (g) | NC 006350 |  | * | * | * |  | 4.07 | B |
| C. tepidum | Chlorobi | NC 002932 |  | * | * | * |  | 2.15 | B |
| C. jeikeium | Actinobacteria | NC 007164 |  | * | * | * |  | 2.46 | B |
| P. marinus | Cyanobacteria | NC 007577 |  | * | * | * |  | 1.71 | B |
| W. endosymbiont | Proteobacteria (a) | NC 006833 |  | * | * | * |  | 1.08 | B |
| B. pseudomallei chr.2 | Proteobacteria (g) | NC 006351 |  | * | * |  |  | 3.17 | B |
| B. subtilis | Firmicutes | NC 000964 | * | * |  | * | * | 4.22 | B |
| E. coli | Proteobacteria (g) | NC 000913 | * | * |  | * | * | 4.64 | B |
| H. pylori | Proteobacteria (e) | NC 000921 | * | * |  | * |  | 1.64 | B |
| D. kamchatkensis | Crenarcheota | NC 011766 |  |  | * |  |  | 1.37 | B |
| D. thermophilum | Dictyoglomi | NC 011297 |  |  | * |  |  | 1.96 | B |
| E. sibricum | Firmicutes | NC 010556 |  |  | * |  |  | 3.03 | B |
| H. aurantiacus | Chloroﬂexi | NC 009972 |  |  | * |  |  | 6.35 | B |
| Hydrogenobaculum | Aquiﬁcae | NC 011126 |  |  | * |  |  | 1.56 | B |
| N. maritimus | Thaumarchaeota | NC 010085 |  |  | * |  |  | 1.65 | B |
| A. laidlawii | Termicutes | NC 010163 |  |  | * |  |  | 1.50 | B |
| H. inﬂuenzae | Proteobacteria (g) | NC 000907 | * |  |  |  |  | 1.83 | B |
| M. genitalium | Tenericutes | NC 000908 | * |  |  |  |  | 0.58 | B |
| Synechocystis | Cyanobacteria | NC 000911 | * |  |  |  | * | 3.57 | B |
| M. penumoniae | Tenericutes | NC 000912 | * |  |  |  |  | 0.82 | B |

Table S3. Measured properties of benchmark datasets. Summary of measured error rates in nine previously published datasets for benchmarking.

| Dataset  Name | Label | | Number | Indel Rate | Subst. Rate | Sum | Avg. Length | Std. Dev. Length |
| --- | --- | --- | --- | --- | --- | --- | --- | --- |
| 454.8079678d | 0.0% | | 98974 | 0.0000 | 0.0000 | 0.0000 | 312.9 | 22.7 |
| 454.807ac35b | 0.2% | | 98974 | 0.0023 | 0.0000 | 0.0023 | 313.5 | 22.8 |
| 454.807a219d | 0.5% | | 98974 | 0.0049 | 0.0000 | 0.0049 | 314.3 | 22.9 |
| 454.8078c401 | 2.8% | | 98974 | 0.0286 | 0.0000 | 0.0286 | 318.2 | 23.1 |
| Sanger.8086f5b2 | 0 | | 84834 | 0 | 0 | 0 | 700.2 | 100.2 |
| Sanger.8022926c | 1.5 × 10^-5^ | | 84834 | 0.000006 | 0.000010 | 0.000015 | 700.3 | 100.0 |
| Sanger.80234712 | 1.5 × 10^-4^ | | 84834 | 0.00006 | 0.00009 | 0.00015 | 700.2 | 99.9 |
| Sanger.801c9689 | 1.5 × 10^-3^ | | 84834 | 0.0006 | 0.0009 | 0.0015 | 700.2 | 99.9 |
| Sanger.801d790f | 1.5 × 10^-2^ | 84834 | | 0.0060 | 0.0090 | 0.0150 | 700.3 | 100.0 |

Table S4. Number and length of predicted genes in 315bp benchmark datasets with simulated 454-style errors. Insertion/deletion errors in the test data cause a dramatic reduction in the number and length of predicted coding regions. Very low predicted coding fractions may indicate poor sequence quality.

| Number of features predicted per sequence: | | | | | | |
| --- | --- | --- | --- | --- | --- | --- |
| Dataset | REFSEQ | FGS | MGA | MGM | ORPH3 | PROD |
| 0.00% | 1.066 | 1.058 | 1.086 | 1.056 | 0.857 | 1.040 |
| 0.20% | 1.066 | 1.057 | 1.142 | 1.088 | 0.816 | 1.059 |
| 0.50% | 1.065 | 1.059 | 1.153 | 1.089 | 0.759 | 1.053 |
| 2.80% | 1.065 | 0.993 | 0.832 | 0.744 | 0.437 | 0.883 |
|  |  |  |  |  |  |  |
| Coding fraction: | | | | | | |
| 0.00% | 0.862 | 0.901 | 0.850 | 0.848 | 0.693 | 0.857 |
| 0.20% | 0.861 | 0.896 | 0.776 | 0.773 | 0.621 | 0.788 |
| 0.50% | 0.858 | 0.886 | 0.701 | 0.702 | 0.548 | 0.734 |
| 2.80% | 0.848 | 0.857 | 0.371 | 0.378 | 0.272 | 0.564 |
|  |  |  |  |  |  |  |
| Average fragment lengths | | | | | | |
| 0.00% | 253.2 | 266.6 | 244.9 | 251.2 | 252.9 | 257.9 |
| 0.20% | 253.2 | 265.6 | 213.1 | 222.7 | 238.7 | 233.3 |
| 0.50% | 253.3 | 262.9 | 191.2 | 202.4 | 226.8 | 219.2 |
| 2.80% | 253.5 | 274.6 | 141.7 | 161.7 | 198.4 | 203.3 |

Table S5. Number and length of predicted genes in 700 bp benchmark datasets with simulated Sanger-style errors. Substitution errors similarly reduce the number and length of predicted coding regions.

| Number of features predicted per sequence: | | | | | | |
| --- | --- | --- | --- | --- | --- | --- |
| Dataset | REFSEQ | FGS | MGA | MGM | ORPH3 | PROD |
| 0.00% | 1.418 | 1.326 | 1.425 | 1.416 | 0.938 | 1.428 |
| 1.50E-005 | 1.422 | 1.334 | 1.430 | 1.422 | 0.942 | 1.438 |
| 1.50E-004 | 1.417 | 1.329 | 1.438 | 1.429 | 0.942 | 1.443 |
| 0.15% | 1.417 | 1.333 | 1.537 | 1.535 | 0.959 | 1.566 |
| 1.50% | 1.419 | 1.216 | 1.767 | 1.832 | 0.921 | 2.040 |
| Coding fraction: | | | | | | |
| 0.00% | 0.870 | 0.908 | 0.860 | 0.870 | 0.447 | 0.874 |
| 1.50E-005 | 0.869 | 0.908 | 0.860 | 0.870 | 0.447 | 0.875 |
| 1.50E-004 | 0.870 | 0.908 | 0.857 | 0.868 | 0.447 | 0.872 |
| 0.15% | 0.870 | 0.906 | 0.829 | 0.845 | 0.441 | 0.853 |
| 1.50% | 0.871 | 0.907 | 0.606 | 0.661 | 0.362 | 0.743 |
| Average fragment lengths | | | | | | |
| 0.00% | 429.6 | 479.7 | 422.5 | 430.1 | 334.0 | 428.3 |
| 1.50E-005 | 428.3 | 476.8 | 421.2 | 428.4 | 332.6 | 426.0 |
| 1.50E-004 | 429.7 | 478.3 | 417.5 | 425.5 | 332.6 | 422.9 |
| 0.15% | 430.1 | 476.1 | 377.7 | 385.5 | 322.5 | 381.4 |
| 1.50% | 429.8 | 442.1 | 240.0 | 252.5 | 275.6 | 255.0 |

Table S6. Command-line parameters used for the models evaluated. All of these programs run in a Linux environment. Prodigal and FGS are open source.

| Tool | Command |
| --- | --- |
| FGS3 | run FragGeneScan.pl -genome-in.fna -out=out -complete=0 -train=454 30 |
| FGS5 | run FragGeneScan.pl -genome=in.fna -out=out -complete=0 -train=illumina 5 |
| MGA | mga -m in.fna > out.mga |
| MGM | gmhmmp -f g -m MetaGeneMark v1.mod -o out.gff in.fna |
| OPH3 | orphelia -s in.fna -m Net300 -o out |
| OPH7 | orphelia -s in.fna -m Net700 -o out |
| PROD | prodigal -i in.fna -f gff -o out |

Note: The following license is to be removed before publication.

The submitted manuscript has been created by UChicago Argonne, LLC, Operator of Argonne National Laboratory (``Argonne"). Argonne, a U.S. Department of Energy Office of Science laboratory, is operated under Contract No. DE-AC02-06CH11357. The U.S. Government retains for itself, and others acting on its behalf, a paid-up nonexclusive, irrevocable worldwide license in said article to reproduce, prepare derivative works, distribute copies to the public, and perform publicly and display publicly, by or on behalf of the Government.
