# Supplementary material for: Short-read reading-frame predictors are not created equal: sequence error causes loss of signal
Source: BMC Bioinformatics. 2012 Jul 28;13:183. doi: 10.1186/1471-2105-13-183 (PMC3526449; doi:10.1186/1471-2105-13-183)
Supplement: Additional file 3 — Supplemental Figures. [file 1471-2105-13-183-S3.doc]

**Figure S1** **Sequencing errors cause marked decreases in reading frame accuracy on the nine benchmark datasets**. Insertion and deletion errors hamper accuracy of gene calls more than do substitution errors at the same rate. The error bars represent the standard deviation of the accuracy between the 14 species included in the benchmark set.

**Figure S2** **Predicted coding fraction as a function of position in read for six artiﬁcial datasets**. The black line is proportional to the sequence length histogram; the style of line indicates the error rate, while the color indicates the gene caller. (A) Predicted coding fraction for three diﬀerent insertion-deletion rates in 315 bp fragments. (B) Predicted coding fraction for two substitution rates in 700 bp fragments. The annotated coding fraction for this artiﬁcial data is 89%, independent of position in the read.

**Figure S3** **Illustration of ab initio annotations on one fragment.** (A) The fasta header for an error-free artiﬁcial fragment from E. sibricum that was annotated as “noncoding” in the center of the fragment. Approximately 12% of artificial shotgun fragments look like this. (B) Refseq annotations show two genes overlap with this fragment: exig 0426(GCN-related N-acetyltransferase) and exig 0427(acireducasedioxygenase ARD). (C) All ﬁve gene prediction algorithms make identical predictions for this error-free fragment, correctly predicting the end of one gene and the beginning of another. The alignment-based evaluation technique counts this fragment as two true positives (since both predicted fragments have protein alignments to the annotated genes), while the reading-frame technique counts this fragment as a single true negative.

**Figure S4** **The 7x7 confusion matrix for reading frame accuracy evaluation.** In addition to the false positive (FP), false negative (FN), true positive (TP), and true negative (TN) categories shared with the binary classiﬁcation problem, gene callers can identify the wrong reading frame (WF) for a fragment. Here 1–6 label reading frames, and NC represents noncoding sequence.


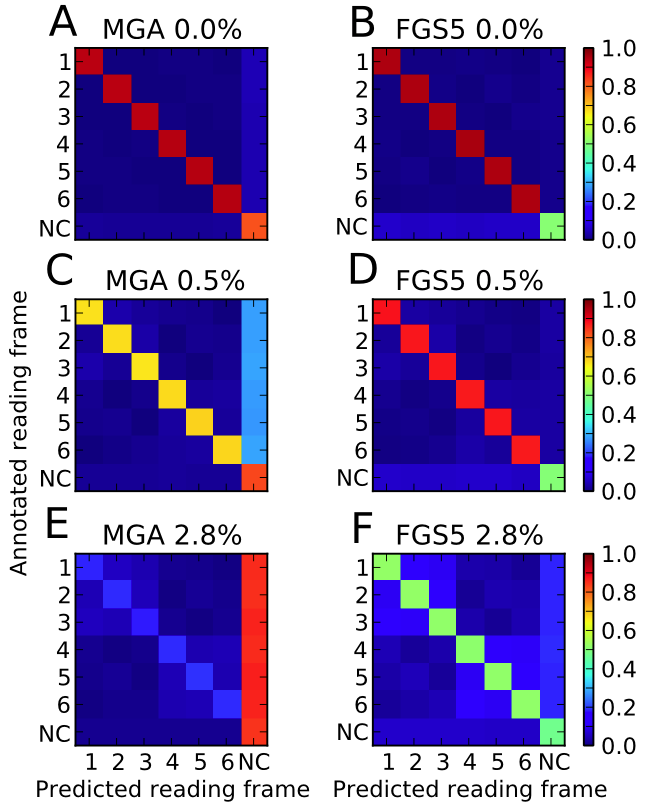


**Figure S5** **Confusion matrices for gene callers with error-containing data.** These heatmaps illustrate the fraction of reads with given annotations that were predicted in each of the seven reading frames. MGA and FGS5 are compared for the three datasets with 0.0%, 0.5%, and 2.8% insertion/deletion errors in 317bp fragments. Values along the diagonal indicate correct predictions while off-diagonal values are erroneous predictions. A) MGA on error-free data. The value in the lower-right cell is the true negative rate for MGA, 75%. B) FGS5 on error-free data. Note the lower true negative rate, corresponding to low specificity. C) MGA on data with 0.5% indel errors. MGA correctly classifies 65% of the coding regions and incorrectly predicts most of the remainder as noncoding. D) FGS on 0.5% errors. E) On data with high error rates, MGA classifies most sequences as noncoding. F) FGS recovers the reading frame about 50% of the time, even in the presence of frameshift-disrupting errors.
